# The activation of G protein-coupled estrogen receptor induces relaxation via cAMP as well as potentiates contraction via EGFR transactivation in porcine coronary arteries

**DOI:** 10.1371/journal.pone.0191418

**Published:** 2018-01-23

**Authors:** Xuan Yu, John N. Stallone, Cristine L. Heaps, Guichun Han

**Affiliations:** 1 Veterinary Physiology & Pharmacology, College of Veterinary Medicine and Biomedical Sciences, Texas A&M University, College Station, TX, United States of America; 2 Women's Health Division, Michael E. DeBakey Institute Texas A&M University, College Station, TX, United States of America; Seconda Universita degli Studi di Napoli, ITALY

## Abstract

Estrogen exerts protective effects against cardiovascular diseases in premenopausal women, but is associated with an increased risk of both coronary heart disease and stroke in older postmenopausal women. Studies have shown that activation of the G-protein-coupled estrogen receptor 1 (GPER) can cause either relaxation or contraction of arteries. It is highly likely that these dual actions of GPER may contribute to the seemingly paradoxical effects of estrogen in regulating coronary artery function. The objective of this study was to test the hypothesis that activation of GPER enhances agonist-stimulated porcine coronary artery contraction via epidermal growth factor receptor (EGFR) transactivation and its downstream extracellular signal-regulated kinases (ERK1/2) pathway. Isometric tension studies and western blot were performed to determine the effect of GPER activation on coronary artery contraction. Our findings demonstrated that G-1 caused concentration-dependent relaxation of ET-1-induced contraction, while pretreatment of arterial rings with G-1 significantly enhanced ET-1-induced contraction. GPER antagonist, G-36, significantly inhibited both the G-1-induced relaxation effect and G-1-enhanced ET-1 contraction. Gallein, a Gβγ inhibitor, significantly increased G-1-induced relaxation, yet inhibited G-1-enhanced ET-1-mediated contraction. Similarly, inhibition of EGFR with AG1478 or inhibition of Src with phosphatase 2 further increased G-1-induced relaxation responses in coronary arteries, but decreased G-1-enhanced ET-1-induced contraction. Western blot experiments in porcine coronary artery smooth muscle cells (PCASMC) showed that G-1 increased tyrosine phosphorylation of EGFR, which was inhibited by AG-1478. Furthermore, enzyme-linked immunosorbent assays showed that the level of heparin-binding EGF (HB-EGF) released by ET-1 treatment increased two-fold; whereas pre-incubation with G-1 further increased ET-1-induced HB-EGF release to four-fold over control conditions. Lastly, the role of ERK1/2 was determined by applying the MEK inhibitor, PD98059, in isometric tension studies and detecting phospho-ERK1/2 in immunoblotting. PD98059 potentiated G-1-induced relaxation response, but blocked G-1-enhanced ET-1-induced contraction. By western blot, G-1 treatment decreased phospho-ERK1/2, however, in the presence of the adenylyl cyclase inhibitor, SQ22536, G-1 significantly increased ERK1/2 phosphorylation in PCASMC. These data demonstrate that activation of GPER induces relaxation via cAMP as well as contraction via a mechanism involving transactivation of EGFR and the phosphorylation of ERK1/2 in porcine coronary arteries.

## Introduction

Cardiovascular disease continues to claim the lives of more women in the U.S. than any other health problem, with coronary heart disease (CHD) as the number one cause of mortality [1]. In early menopause, estrogen therapy helps protect against CHD [2, 3]; yet paradoxically, estrogen is associated with an increased risk of both CHD and stroke in postmenopausal women >65 years of age [4]. Therefore, effective prevention and treatment of CHD in this older population remains ambiguous.

Evidence from the literature suggests that GPER plays an important role in mediating cardiovascular actions of estrogen [5], however, the current understanding of the underlying mechanisms are limited and largely contradictory. For example, the vasodilatory effect of GPER activation has been demonstrated in rat carotid, rat aorta, human mammary arteries, and porcine coronary arteries [6–11]. On the other hand, it has also been reported that GPER mediates a vasoconstrictive effect in isolated perfused rat kidneys [12]. Previously, we reported that one of the mechanisms by which activation of GPER regulates coronary artery relaxation was via cAMP/PKA-dependent activation of MLCP [13]. Additionally, our previous studies suggest that GPER-mediated coronary artery relaxation was also mediated via a novel downstream signaling pathway of cAMP, Epac (exchange proteins directly activated by cAMP), in parallel with cAMP/PKA [13, 14]. Thus, GPER has been shown to mediate opposite effects on arteries from different vascular beds. Based on these findings, we propose that these dual actions of GPER may contribute to the seemingly paradoxical effects of estrogen in regulating coronary artery function. However, the mechanism underlining the GPER-mediated vasoconstriction is not clear.

In human coronary arterioles, estrogen potentiated angiotensin II-induced vasoconstriction via GPER and EGFR activation [15]. The potent constrictive effect of GPER activation was shown in an isolated rat kidney perfusion study. Following pre-constriction, addition of the GPER agonist G-1 caused renal artery relaxation, however, in the absence of pre-constriction, G-1 alone caused arterial contraction which was blocked by inhibitors of EGFR and MEK kinase [12, 16]. These data indicate that the activation of GPER induces transactivation of EGFR. Filardo et al. [17] also provided evidence of GPER-mediated transactivation of EGFR. They reported that in MCF-7, SKBR3 and MDA-MB-231 cells, estrogen caused attenuation of ERK1/2 activity via GPER-mediated Gβγ-dependent transactivation of EGFR. However, the mechanism by which GPER mediates the opposing effects of estrogen and whether GPER contributes to the paradoxical effects of estrogen in postmenopausal women remain unknown. Endothelin-1 (ET-1) is a well-accepted vaso-constrictor for inducing coronary constriction [18]. Therefore, in this study, the hypothesis that activation of GPER enhances agonist ET-1-induced coronary artery contraction through transactivation of EGFR and phosphorylation of ERK1/2 was tested.

## Methods and materials

### Materials

Antibodies used in immunoblotting were: p-ERK1/2 from Cell Signaling (Cat#: 9101, 1:1000 dilution), p-EGFR from Santa Cruz (Cat#: SC-12351, 1:500 dilution) and β-actin from Novus Biologicals (Cat#: NB600-501, 1:2000 dilution). G-1 and G36 were purchased from Azano Pharmaceuticals Incorporation. Inhibitors used in the isometric tension studies were purchased from Tocris Bioscience. All other chemicals were purchased from Sigma Aldrich.

### Supply of coronary arteries

Porcine hearts were obtained from an approved and regulated supplier; the geographic coordinates are latitude 30.372080° and longitude -96.070557°. The hearts were placed into ice-cold Krebs buffer of the following composition: 131.5 mM NaCl, 5 mM KCl, 1.2 mM NaH_2_PO_4_, 2.5 mM MgCl_2_, 1.2 mM CaCl_2_, 2 g glucose and 2 g NaHCO_3_ in 1 L with pH 7.4 (The solution was previously oxygenated with 95% O_2_-5% CO_2_ for 30 minutes). Hearts were kept on ice during transport to the laboratory, where coronary arteries were dissected as described previously [11, 13].

### Isometric tension studies

The left anterior descending (LAD) coronary arteries were dissected and cleaned of excess connective tissue and fat. To eliminate indirect effects of endothelium-derived vasoactive factors, the endothelium was removed physically by rubbing the intimal surface and tested by observing the absence of bradykinin-induced relaxation of PGF2α-induced contraction. The LAD was cut into ~3 mm rings and mounted on two parallel tissue supports in the tissue incubation chamber, with one support fixed to the stationary chamber and the other attached to a force transducer (AD Instruments). Isometric contractile force was recorded on a computer using LabChart software. The tissue bathing solution was Krebs buffer, oxygenated continuously with 95% O_2_-5% CO_2_ and maintained at 37°C. Coronary arterial rings were equilibrated for 90 min under resting tension of 20 mN, and the bath solution was changed every 30 min. For relaxation response experiments, at the plateau of ET-1 (10 nM)-induced contraction, inhibitors were added 30 min prior to the measurement of a complete G-1 concentration-response relationship (1 to 3000 nM). Relaxation responses were calculated as the percent reduction in tension from ET-1 pre-contracted state. For vasoconstriction response, G-1 and inhibitors were also used to pretreat the arterial rings before adding endothelin 1 (ET-1, 0.1–30 nM) to induce a contractile response. Arterial rings were collected, oven-dry, and weighed after experiments. Contractile responses were calculated as force generated by arterial rings calibrated by artery dry weight with unit mN/mg.

### Cell culture

We adopted the method of dispersing coronary artery cells by Chamley-Campbell et al. with some modification [13, 19, 20]. Briefly, coronary arteries were dissected, cut into pieces and placed into 3 mg/ml collagenase in a modified dissociation medium (110 mM NaCl, 5 mM KCl, 2 mM MgCl_2_, 0.16 mM CaCl_2_, 10 mM HEPES, 10 mM NaHCO_3_, 0.5 mM KH_2_PO_4_, 0.5 mM NaH_2_PO_4_, 0.49 mM EDTA, 10 mM Taurine 10 mM glucose and 15% fetal bovine serum (Lonza)) at 37°C for two hours. Then the tissue pieces were dispersed into single cell with complete SmBM basal medium (Lonza) and seeded into 60-mm gelatin-coated plastic dishes with density of 2x10^5^ cells/ml. Primary cultured porcine coronary artery smooth muscle cells (PCASMCs) were maintained in complete SmBM basal medium. Cell cultures were kept at 37°C and under 5% CO_2_ in a humidified incubator. The purity of PCASMCs was verified by positive staining with smooth muscle-specific α-actin [21]. Primary PCASMCs were cultured to 80% -90% confluence, and then we employed passage 4–5 for biochemical experiments.

### Western blot

Coronary artery rings were collected after various drug treatments in isometric tension studies and were snap-frozen in liquid nitrogen. The tissues were pulverized and then lysed in RIPA lysis buffer (Sigma) with protease and phosphatase inhibitors. PCASMCs were harvested and lysed in the same lysis buffer. Protein concentrations were determined by BCA protein assay (Pierce) and samples were separated on precast 4–12% Bis-Tris gels (Invitrogen) according to the manufacturer’s instructions. Then proteins were transferred to PVDF membrane (EMD Millipore) at 100 V for two hours. Membranes were blocked with 5% non-fat milk for one hour at room temperature and then incubated with primary antibodies in TBST, a mixture of tris-buffered saline (TBS) and Tween 20 (0.1%), containing 5% non-fat milk for overnight at 4°C. After washing and incubating with secondary antibodies, protein bands were detected with chemiluminescence. Then the membranes were stripped by washing in stripping buffer (Thermo Scientific), and then probed with β-actin antibody to be used as protein loading controls.

#### Enzyme-linked immunosorbent assay

The level of heparin binding epidermal growth factor (HB-EGF) in the PCASMCs samples was measured by enzyme-linked immunosorbent assay, according to manufacturer’s instructions (ThermoFisher Scientific). Briefly, PCASMCs, passage 4–5, were serum-deprived for 24 hours after cells reached approximately 50–60% confluence. After incubation with different drugs, cell culture media was collected in clean tubes and centrifuged at 3000 rpm for 20 min. The supernatants were kept for assay in duplicates. The optical density of each sample was measured by using a microplate reader at 450 nm and 550 nm. The average absorbance (450 nm minus 550 nm) of each sample was calculated from the standard curve, and the concentration of HB-EGF was expressed as pg/ml.

### Cyclic AMP assay

Cyclic cAMP production was measured with Cayman’s cAMP assay, which is competitive enzyme-linked immunosorbent assay (ELISA) (Cayman Chemical), as described previously (78). Briefly, PCASMCs (passages 4) were cultured in culture dishes (35-mm) in SmGM medium for three days. After reaching 85–90% confluence, cells were serum-deprived for 18 h in phenol red-free α-MEM buffer with 3-isobutyl-1-methylxanthine (100 μM) to inhibit phosphodiesterases. Then cells were treated with G-1 (1 μM), or ET-1 (10 nM), 0.1% DMSO was used as solvent control.

### Statistics

All data were analyzed using GraphPad Prism (GraphPad Software Inc., San Diego, CA). In isometric tension studies, the concentration-responses of G-1 and ET-1 with or without inhibitors were analyzed by two-way analysis of variance (ANOVA). For immunoblotting experiments and ELISA, one-way ANOVA was used. Bonferroni correction was used as post-hoc test to correct type 1 errors associated with ANOVA in all data sets. All data were expressed as means ± SEM. P value ≤0.05 indicated a significant difference.

## Results

### GPER and coronary artery tension regulation

**Fig 1:**
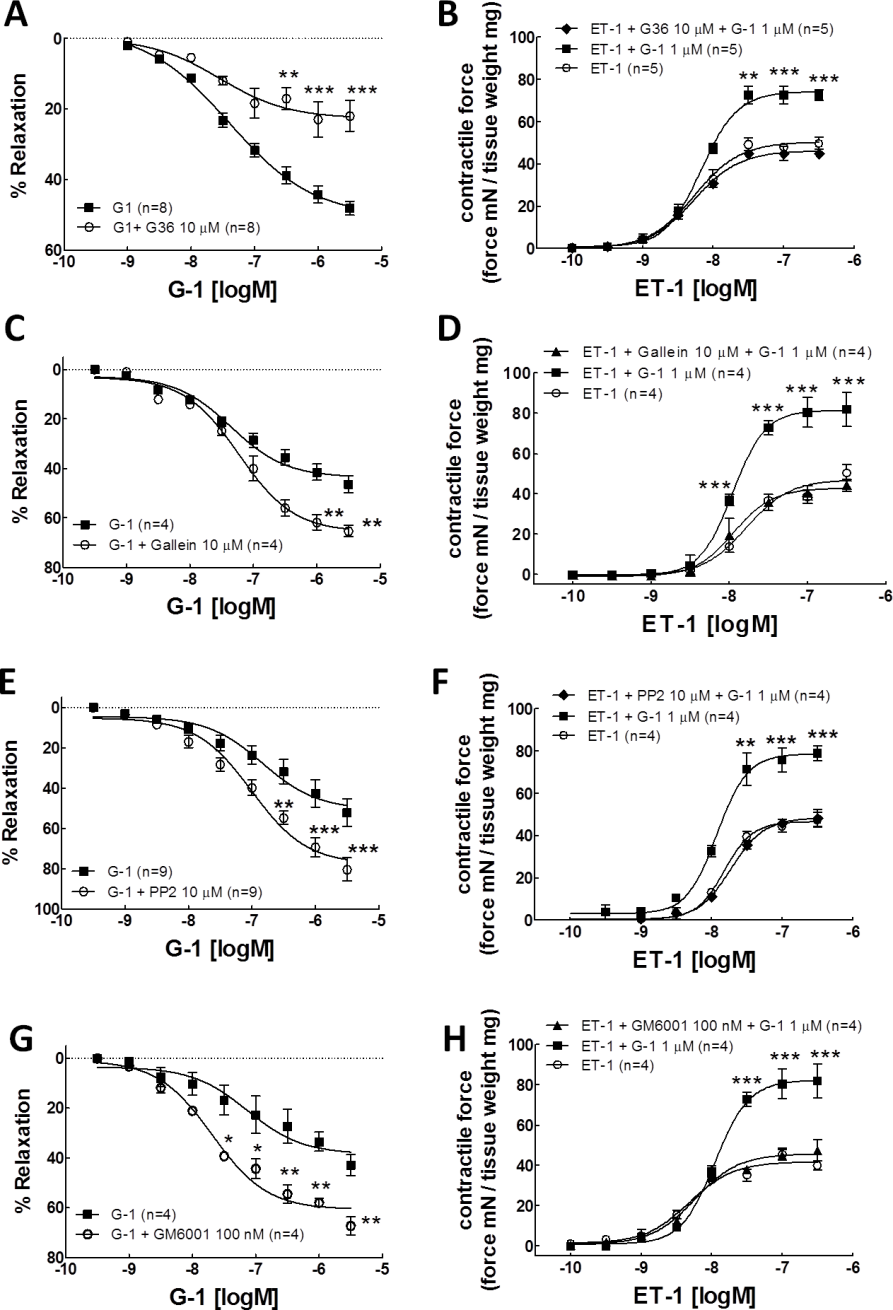
G-1 causes coronary artery relaxation but also potentiates ET-1-induced coronary artery contraction via Gβγ/Src/MMP pathway. Each number of experiments represents the mean result of arterial rings from one porcine heart. A. Concentration-response relationship for G-1-induced coronary relaxation with or without the GPER antagonist, G36. B. Concentration-response relationship for ET-1-induced contraction following pretreatment with G-1 or G-1+G36. C: concentration-response relationship for G-1-induced porcine coronary artery relaxation in the presence or absence of selective Gβγ inhibitor, gallein (10 **μ**M). D: concentration-response relationship for ET-1-induced contraction in the presence of G-1 or G-1+gallein. E: concentration-response relationship for G-1-induced porcine coronary artery relaxation in the presence or absence of selective inhibitor for Src-family kinases, PP2 (10 μM). F: concentration-response relationship for ET-1-induced contraction in the presence of G-1 or G-1+PP2. G: concentration-response relationship for G-1-induced porcine coronary artery relaxation in the presence or absence of MMP inhibitor, GM6001 (100 nM). H: concentration-response relationship for ET-1-induced contraction in the presence of G-1 or G-1+GM6001. In A, C, E, and G, each point represents the mean relaxation response ± SEM. *P < 0.05, **P < 0.01, ***P<0.001, compared with G-1 group using two-way ANOVA. In B, D, F, H, each point represents the mean developed tension ± SEM. Developed tension was the force generated by artery rings normalized to arterial dry weight (mN/mg). **P < 0.01, ***P<0.001, compared with ET-1 group using two-way ANOVA.

Previously we have reported that activation of GPER by G-1, a potent and selective GPER agonist [22], caused endothelium-denuded coronary artery relaxation after PGF2α pre-contraction [11, 13]. In this study, endothelium-denuded LAD coronary artery responses to G-1 under ET-1 stimulation were tested. First, the arterial rings were pre-contracted with ET-1 (10 nM). After the ET-1-mediated pre-contraction response reached steady state, G-1 was added cumulatively to construct a concentration-response relaxation curve. In some experiments, after ET-1-induced contraction reached steady state, arterial rings were pretreated with G36 (10 μM), a highly selective GPER antagonist [23], prior to G-1-induced relaxation. As shown in Fig 1A, G-1 clearly induced a concentration-dependent relaxation response compared to the vehicle control group, with EC_50_ at 0.034 μM and maximum relaxation of 46.4 ± 3.4%; and the relaxation response was significantly inhibited by G36, without changing EC_50_ of G-1 (Table 1). Next, we tested whether GPER enhances coronary contraction by applying ET-1 (0.1–30 nM) cumulatively to construct a concentration-response curve in the presence of G-1 with or without G36. As shown in Fig 1B, pre-incubation with G-1 (1 μM), a concentration that induces almost maximum binding to GPER without overlapping with ERα and ERβ [22], significantly increased ET-1-induced concentration-dependent contraction, compared to the vehicle (DMSO) pre-incubation group. G36 (10 μM) completely blocked the enhanced ET-1 contraction observed after pretreatment with G-1 alone. The maximal contractile force of ET-1 in G-1 and G-1+G36 pretreated artery rings were 79.62 +7.48 and 45.0 ± 1.6 mN/mg without changing EC_50_ of ET-1 (8.6 nM) (Table 2). These findings suggest that activation of GPER relaxes coronary artery under condition of pre-contraction, but also potentiates ET-1-induced coronary vasoconstriction when arterial rings are pre-incubated with G-1.

**Table 1:** Effects of Gβγ/EGFR/ERK1/2 signaling compounds on porcine coronary artery relaxation response to 3 μM G-1.

compounds	% increase of the G-1 relaxation	% relaxation		P value	EC_50_ (μM)
DMSO^+^			46.39±3.37 (n = 10)		0.034
G36 10 μM			21.95±4.38 (n = 8)	P<0.001	0.035
Gallein 10 μM	40.96%		65.39±2.25 (n = 4)	P<0.01	0.017
GM6001 0.1 μM	44.99%		67.26±3.62 (n = 4)	P<0.01	0.034
AG1478 5 μM	50.21%		69.68±3.52 (n = 8)	P<0.001	0.034
PP2 10 μM	73.36%		80.42±5.77 (n = 6)	P<0.001	0.010
PD98059 1 μM	39.82%		64.86±3.12 (n = 6)	P<0.01	0.033

Values are given as mean relaxation effect ± SEM. Each n represents data averaged from arterial rings of one porcine heart indicated in parentheses. Arterial rings were pretreated with each of the inhibitors and the results were compared to rings studied in the absence of inhibitors (DMSO^+^). P<0.01, P<0.001, significant difference compared with rings studied in the absence of inhibitors (DMSO^+^) by using two-way ANOVA.

**Table 2:** Effects of compounds on ET-1 (0.1–30 nM) concentration contraction responses in porcine coronary artery.

compounds	Contractile force (mN/mg)	P value	EC_50_ (nM)
ET-1^+^	49.83±2.96		8.59
G-1 1 μM	79.62±7.48 (n = 6)	P<0.001	8.59
G36 10 μM+G-1 1 μM	44.99±1.62 (n = 6)	P<0.001	8.61
Gallein 10 μM+G-1 1 μM	44.22±2.87 (n = 4)	P<0.001	8.59
GM6001 0.1 μM+G-1 1 μM	47.51±5.26 (n = 4)	P<0.001	8.63
AG1478 5 μM+G-1 1 μM	30.14±4.21 (n = 6)	P<0.001	8.58
PP2 10 μM+G-1 1 μM	48.30±3.95 (n = 5)	P<0.001	8.59
PD98059 1 μM+G-1 1 μM	49.45±4.82 (n = 4)	P<0.001	8.57

Values are given as mean contractile force ± SEM. The number of experiments is indicated in parentheses. Arterial rings were pretreated with each of the inhibitors and the results were compared to ET-1 in the absence of inhibitors (^+^ET-1). P<0.001, significant difference compared with ET-1 in the absence of inhibitors (^+^ET-1) by using two-way ANOVA.

### The role of Gβγ in coronary artery tension regulation

The activation of GPER as a stimulus for increased Gβγ signaling has been implicated in MCF-7, SKBR3 and MDA-MB-231 cell lines [17], however, there is no direct evidence showing GPER mediates Gβγ signaling in coronary arteries. Therefore, the functional role of Gβγ in GPER-mediated coronary artery reactivity was tested. The specific Gβγ inhibitor, gallein [24], was used to block Gβγ signaling in LAD arterial rings in both G-1 relaxation and ET-1 contractile responses. Gallein (10 μM) pretreatment further increased G-1-induced relaxation of coronary arteries which were pre-constricted with ET-1 (10 nM), with maximal relaxation of 65.4 ± 2.3%; and the EC_50_ of G-1 increased from 0.034 μM in the absence of gallein to 0.017 μM in the presence of gallein (Fig 1C, Table 1). These data suggest that there is a G-1-induced contraction component which is mediated by Gβγ and masked by the more robust relaxation effect of G-1. To further confirm this notion, gallein was used in the experiments of ET-1-induced contraction in the presence of G-1. As expected, gallein (10 μM) pretreatment of the artery rings completely blocked G-1-induced enhancement of ET-1 vasoconstriction, returning ET-1 concentration-response curve to levels not significantly different from control levels, the maximal contractile force was from 79.62 +7.48 mN/mg in the presence of G-1 down to 44.2 ± 2.9 mN/mg with the addition of gallein (Fig 1D, Table 2). These findings suggest that activation of GPER by G-1 induces coronary artery relaxation as well as contraction and Gβγ is involved in the GPER-mediated coronary artery contractile response.

### The role of Src in coronary artery tension regulation

Src is a non-receptor tyrosine kinase and a key element in GPER transactivation of growth factor receptor signaling transduction in a variety of cell lines [25]. Evidence has shown that Src modulates coronary artery contractility [26]. Thus, we next explored the role of Src in GPER-mediated potentiation of coronary artery contraction. Arterial rings were first pre-incubated with phosphoprotein phosphatase 2 (PP2, 10 μM), an inhibitor of Src [25] and then both of the cumulative G-1- and ET-1-concentration-response relationships were recorded in separate experiments. As expected, inhibition of Src with PP2 (10 μM), enhanced G-1-induced relaxation response of ET-1-precontracted arterial rings, with EC_50_ of 0.01 μM and maximal relaxation of 80.42 ± 5.77% (Fig 1E, Table 1). PP2 (10 μM) also reversed G-1 (1 μM) -enhanced ET-1-vasoconstriction, maximal constriction force dropping from 79.62 +7.48 to 48.3+3.95 mN/mg without changing EC_50_ of ET-1(Fig 1F, Table 2). These findings suggest that Src is also involved in the GPER-induced potentiation of coronary artery contraction.

#### The role of MMP in coronary artery tension regulation

Matrix metalloproteases (MMP) play an important role in GPCR-mediated HB-EGF shedding and EGFR transactivation. It has also been shown that MMP are involved in pressure-induced myogenic tone mediated by EGFR transactivation in mouse mesenteric resistance arteries [27]. To investigate the role of MMP in the change of artery tension mediated by GPER, GM6001, a MMP inhibitor [28], was used in isometric tension experiments. Pretreatment of coronary artery rings with GM6001 (100 nM) significantly enhanced the relaxation effect of G-1 with maximal relaxation of 67.3 ±3.6%, compared to the maximal relaxation of 46.4±3.4% induced by G-1 alone (Fig 1G, Table 1); but totally blocked G-1 (1 μM) enhanced ET-1-contraction with maximal contraction of 47.51+5.26 (Fig 1H, Table 2). These findings suggest that MMP plays a role in the GPER-mediated tension regulation of coronary artery.

### EGFR transactivation and coronary artery tension regulation

**Fig 2:**
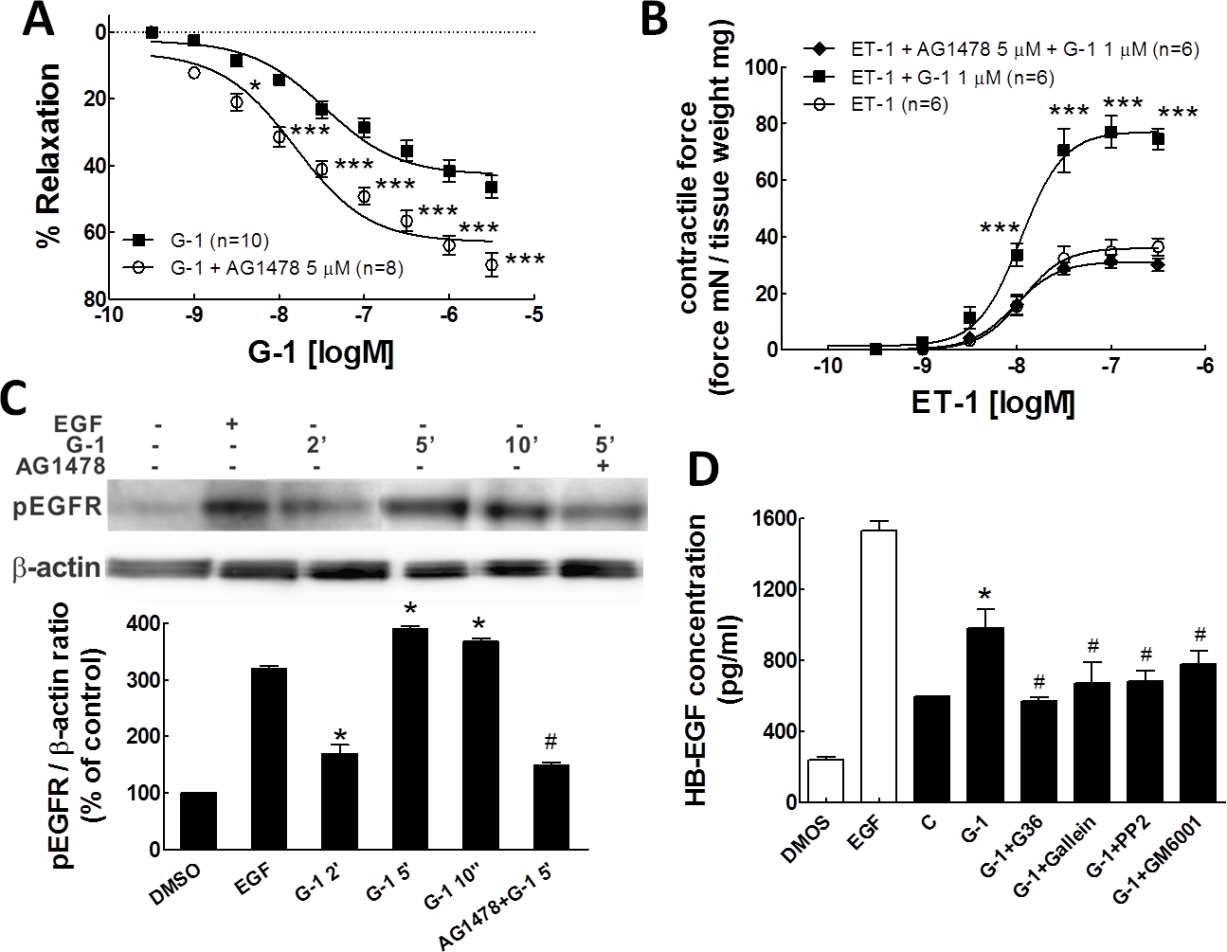
EGFR transactivation is involved in the coronary tone regulation of G-1. A: Concentration-response relationship for G-1-induced porcine coronary artery relaxation in the presence or absence of the selective EGFR inhibitor, AG1478 (5 **μ**M). Each point represents the mean relaxation response ± SEM.*P < 0.05, ***P<0.001, compared with G-1 treatment using two-way ANOVA. B: concentration-response relationship for ET-1-induced contraction in the presence of G-1 or G-1+AG1478. Each point represents the mean developed tension ± SE. Developed tension was the force generated by artery rings normalized to arterial dry weight (unit mN/mg). ***P<0.001, compared with ET-1 group using two-way ANOVA. C: western blot detection of phosphorylated EGFR in PCASMCs. Cells were incubated with DMSO (solvent control), EGF (100 ng/ml) for 10 min, G-1 (1**μ**M) for 2 min, G-1 (1 **μ**M) for 5 min, G-1 (1 **μ**M) for 10 min and AG1478 (5 **μ**M) +G-1 (1 **μ**M) For 5 min. Top: a representative western blot from three individual experiments examining phospho-EGFR levels. Bottom: bar graph of the quantitative data of the western blot bands evaluated by densitometry. Protein amounts were normalized to β-actin, which was employed as a control for protein loading. *P < 0.05, compared to the group as indicated in the graph. D. HB-EGF activity was evaluated in PCASMCs. SMCs were serum deprived for 18 hours before treating with drugs. White bars: Cells were treated for 10 minutes with: 0.1% DMSO as solvent control; and EGF (10 ng/ml) as positive control (n = 3). Black bars: After pre-incubating with ET-1 (10 nM) for 10 min, cells then were treated with 0.1% DMSO, G-1 (100 nM), G-1 (100 nM) + G36 (1 **μ**M), G-1 (100 nM) + Gallein (1 **μ**M), G-1 + PP2 (1 **μ**M), and G-1 (100 nM) + GM6001 (100 nM) (n = 3), * p<0.05, compared to the control group (C); # P < 0.05, compared to G-1 treatment group (G-1) as indicated.

Activation of EGFR has been implicated in many cardiovascular diseases [29]. Although it has been demonstrated that activation of GPER induces EGFR transactivation in breast cancer cells [25], there is not much information available in vascular smooth muscle. We first tested the functional role of EGFR in isometric tension experiments by employing AG1478, a selective EGFR tyrosine kinase inhibitor [30], to construct concentration-response curves of either G-1 induced relaxation or ET-1-induced contraction. Pretreatment of arterial rings with AG1478 (5 μM) augmented G-1-induced relaxation by more than 50% at maximal relaxation (46.4 ± 3.4% to 69.7 ± 3.5%) (Fig 2A, Table 1), without changing G-1 EC_50_ values (0.034 μM). Furthermore, the inhibition of EGFR with AG1478 significantly reversed G-1 (1 μM) enhanced ET-1-induced contractile response (Fig 2B, Table 2). These data indicate that activation of GPER induces EGFR activity that is involved in the G-1 potentiation of vascular contraction.

We then measured the effect of GPER activation on tyrosine phosphorylation of EGFR in PCASMCs by western blot. Our results show that G-1 (1 μM) treatment of PCASMCs significantly increased EGFR tyrosine phosphorylation at 5 and 10 min, to a phosphorylation level similar to that observed with EGF (10 ng/ml), which served as a positive control. Pretreatment with AG1478 (5 μM) significantly attenuated G-1-stimulated tyrosine phosphorylation of EGFR (Fig 2C). These data demonstrate that activation of GPER induces transactivation of EGFR. Filardo et al. have reported that GPER mediates EGFR transactivation and its downstream signaling through the release of surface-associated heparin-binding EGF (HB-EGF) from its precursor protein in breast cancer cells [25]. In order to determine whether HB-EGF release is indeed the cause of G-1-induced activation of the EGFR in porcine coronary artery, the level of HB-EGF release was measured in PCASMCs after different drug treatments. Using an enzyme-linked immunosorbent assay (ELISA) ET-1 (10 nM) significantly increased HB-EGF two-fold from a basal level of 238.7 ± 20.7 pg/ml to 596.9 ± 33.4 pg/ml. In the presence of G-1 (100 nM), the level of HB-EGF stimulated by ET-1 reached 982.3 ± 39.9 pg/ml, a significant four-fold increase over basal levels. Furthermore, the elevated HB-EGF release by G-1 pre-incubation was significantly attenuated by the specific inhibitors previously tested: G36 (10 μM, 573.2 ± 28.2 pg/ml), gallein (1 μM, 670.6 ± 24.5 pg/ml), PP2 (1 μM, 680.1 ± 53.8 pg/ml) or GM6001 (100 nM, 776.0 ± 34.4 pg/ml) (Fig 2D). Together, these results suggest that activation of GPER induces transactivation of EGFR in PCASMC which contributes to the vasoconstriction effect of GPER.

### ERK1/2 activation and coronary artery tension regulation

**Fig 3:**
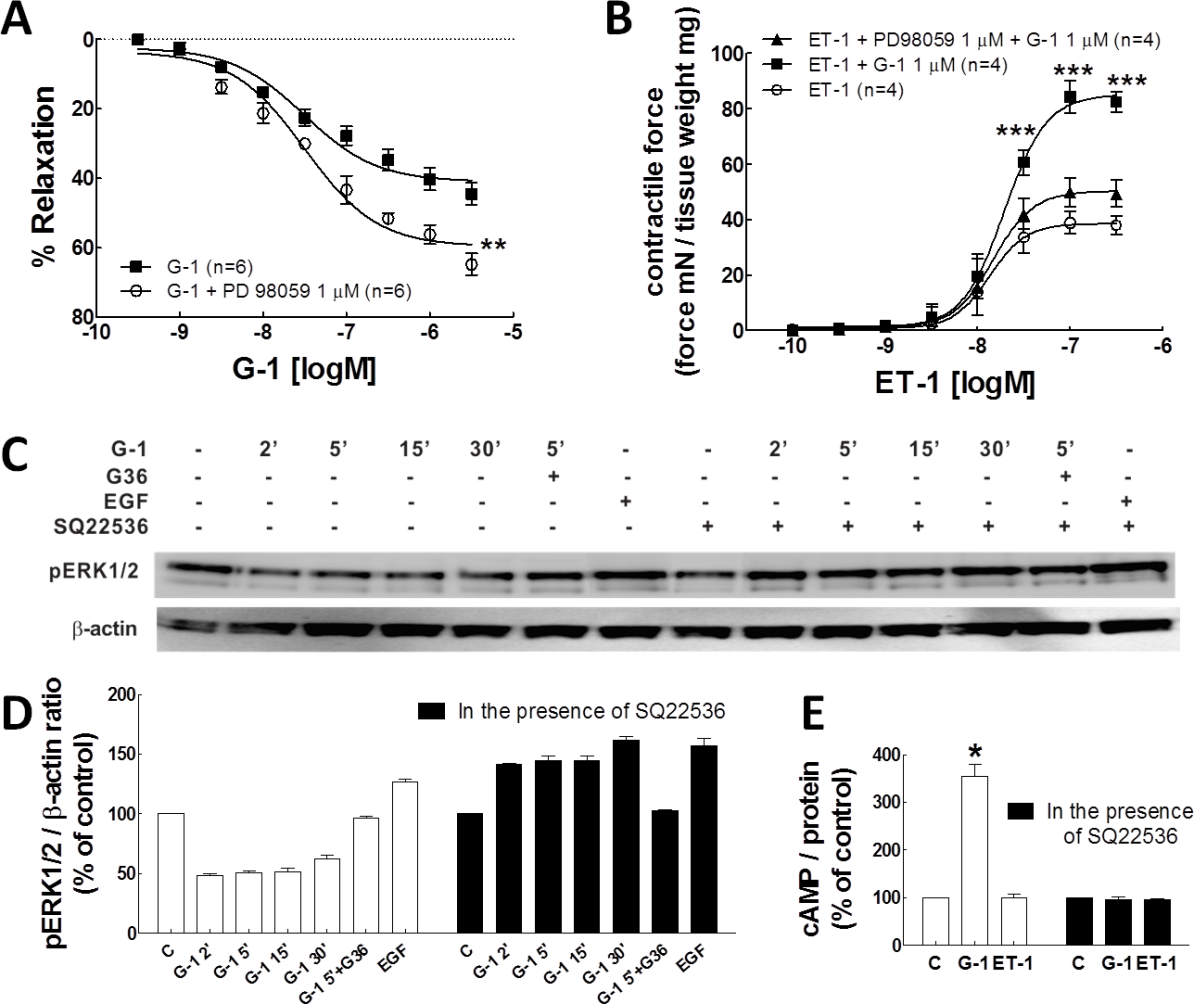
G-1 stimulates ERK1/2 phosphorylation in porcine coronary arteries and isolated SMCs in the presence of adenylyl cyclase inhibition. A: concentration-response relationship for G-1 in the presence or absence of PD98059, a selective inhibitor of MEK kinase. Results are expressed as mean relaxation response ± SEM of six experiments. **P < 0.01, compared with the G-1 group by two-way ANOVA analysis. B: concentration-response relationship for ET-1-induced contraction in the presence of G-1 or G-1+PD98059. Each point represents the mean developed tension ± SEM. ***P<0.001, compared with ET-1 group using two-way ANOVA. C & D: western blot detection of phosphorylation of ERK1/2 in PCASMCs. Cells were pretreated in the presence or absence of adenylyl cyclase inhibitor, SQ22526 (100 μM), then incubated with DMSO (solvent control), G-1 (1 μM, with different collecting time points), G36 (10 μM) + G-1 (1 μM), or EGF (10 ng/ml), a positive control. C: a representative western blot from three individual experiments. D: bar graph of the quantitative data of the western blot bands evaluated by densitometry. Protein amounts were normalized to β-actin, which was employed as a control for protein loading. *, ^+^ and ^#^ are P < 0.05. ^+^ is the comparison of G-1 treatment at the time points indicated with solvent control in the absence of SQ22536; * is the treatment of G-1 in the presence of SQ22536 compared to the same time point of G-1 treatment in the absence SQ22536; ^#^ is the comparison of G-1 treatment with solvent control in the presence of SQ22536. E: cAMP production of the porcine coronary artery smooth muscle cells (SMCs) in response to G-1 or ET-1. Cells were treated with G-1 (1 μM) or ET-1 (10 nM) respectively for 20 mins in the presence or absence of SQ22536 (100 μM). Each point represents the mean production ± SEM. *P<0.05, compared with solvent control (C) group. Results are normalized by total protein amount.

GPER activation can stimulate Src-related tyrosine kinase activity-dependent EGFR transactivation and then cause ERK1/2 phosphorylation in breast cancer cells [25]. Inhibition of ERK1/2 with PD98059, an MEK inhibitor, significantly attenuated G-1-induced vasoconstriction of rat renal arteries [12]. In this study, the role of ERK1/2 was tested by using PD98059 in isometric tension studies and phospho-ERK1/2 detection in PCASMCs by immunoblotting. The pretreatment with PD98059 (1 μM) significantly enhanced G-1-induced relaxation response of arterial rings (Fig 3A, Table 1). Furthermore, inhibition of ERK1/2 with PD98059 significantly reversed G-1-enhanced ET-1-mediated contraction (Fig 3B, Table 2). Our previous work has shown that GPER-induced relaxation is mediated via cAMP signaling [13, 14]. Therefore using western blot analysis, we tested whether the cAMP signaling pathway inhibits phosphor-ERK1/2 by applying the adenylyl cyclase inhibitor, SQ22536, in PCASMCs. The results show that G-1 (1 μM) treatment alone decreased phospho-ERK1/2 significantly in PCASMCs. However, in the presence of the potent adenylyl cyclase inhibitor, SQ22536 (100 μM), G-1 significantly increased ERK1/2 phosphorylation at all of the time points with the highest level at 30 min, similar to treatment with EGF (10 ng/ml) (Fig 3C & 3D). Cyclic AMP essay showed that G-1 (1 μM) significantly increased cAMP production and SQ22536 (100 μM) completely blocked the stimulation effect of G-1. ET-1, however, had no effect on cAMP production (Fig 3E). Taken together with our previous findings [13, 14], these data suggest that activation of GPER induces relaxation via cAMP signaling and contraction via transactivation of EGFR and phosphorylation of ERK1/2, with cAMP-mediated relaxation the predominant response in coronary arteries.

## Discussion

In this study, we have found that GPER may mediate both the relaxation and contraction responses of coronary arteries. The contractile effect of a GPER agonist G-1 was induced through EGFR transactivation and subsequent ERK1/2 activation via Gβγ/Src/MMP signaling pathway.

EGFR plays a key role in myogenic tone regulation as well as G protein-coupled receptor (GPCR)-mediated vasoconstriction response [31–35]. Myogenic tone is a contraction induced in response to changes in vascular transmural pressures; therefore, the development of myogenic tone is important in the regulation of blood pressure. In studies of pressure-induced myogenic tone, the EGFR inhibitor, AG1478, significantly inhibited the mesenteric and coronary arteriole tone development in mice and western blot analysis revealed the myogenic tone was associated with phosphorylation of EGFR tyrosine kinase [31, 32]. Furthermore, the increased mesenteric and coronary arterial myogenic tone in a mouse model of type 2 diabetes was demonstrated to be clearly related to the elevation of EGFR protein expression and phosphorylation [36]. Similarly, the GPCR-mediated vasoconstrictor responses induced by norepinephrine, endothelin-1 (ET-1) and angiotensin II (Ang II) were significantly increased in the mesenteric bed of a rat model of type I diabetes and the treatment of the diabetic animals with AG1478 normalized the altered agonist-induced vasoconstriction responses [37]. Although EGFR transactivation does not seem to be directly involved in the mechanism of inducing the initial contractile force by GPCR agonists, EGFR does play a role in sustained contraction [38]. EGFR activity is also required for aldosterone-enhanced angiotensin II–mediated vasoconstriction [33], but not for the contractile force induced by angiotensin II alone. Interestingly, GPER was reported to be the key upstream target in the EGFR transactivation induced by aldosterone-angiotensin II interaction in human coronary microarteries [15]. Furthermore, estrogen and hydrocortisone also enhanced the angiotensin II contractile response in a similar manner. Aldosterone, 17β-estradiol and hydrocortisone all potentiated contractile effect of angiotensin II; while, G15[39], a GPER antagonist, and AG1478 prevented the potentiation of contraction by these steroids, indicating that GPER plays an important role in EGFR transactivation[15]. It has also reported that comparing the effects of G-1 on the coronary arteries in the hearts isolated from male and female rats, arteries from males demonstrated less relaxation than that from females, due to the elevated superoxide production in the arterial tissues of male rats; however, no signaling pathway was explored [40].

In the current study, we determined that the inhibition of EGFR with AG1478 increased GPER-mediated coronary artery relaxation; but abolished GPER-mediated potentiation of ET-1-induced contraction without any effect on the contractile force induced by ET-1 alone, suggesting that there is a contractile component in the effect of GPER activation that involves EGFR. G-1 stimulation of heparin-binding EGF (HB-EGF) release and tyrosine phosphorylation of EGFR have further confirmed the notion that activation of GPER indeed causes transactivation of EGFR and thus induces enhanced vasoactive contraction of coronary artery. It appears that under condition pre-contraction, G-1-induced vasorelaxation dominates; however, when pre-treat the arterial rings with G-1 before applying vasoconstrictor ET-1, the vasoconstriction effect dominates. Apparently, in the pre-contracted arterial ring experiment, the constriction effect mediated by EGFR transactivation exerted opposing effect on the predominate response of relaxation mediated by cAMP in the action of GPER; when AG1478 was applied to block the EGFR pathway, the opposing vasoconstriction effect was inhibited, therefore, more vasorelaxation response of GPER activation being demonstrated. Vice versa, in the ET-1 concentration-response vasoconstriction experiment, pre-incubation of the arterial rings with G-1 induced more vasoconstriction response to ET-1 through transactivation of EGFR and AG1478 inhibited the vasoconstriction effect of G-1 pre-treatment. Together the evidence provided here suggests that GPER-mediated transactivation of EGFR is an important mechanism in the regulation of vascular tone.

The GPER-mediated transactivation of EGFR occurs via Gβγ-subunit signaling in breast cancer cells [25, 41, 42], following the same pathway as in other GPCRs [43]. Heterotrimeric G-proteins are composed of three protein subunits: α, β, and γ. Following binding of specific ligands to GPCRs, Gα subunit in the activated trimetric G protein dissociates from the Gβγ complex and activates adenylate cyclase, which stimulates the production of cAMP. Whereas, Gβγ signals to its downstream canonical effectors which include adenylyl cyclase, phospholipase Cβ, inwardly rectifying K^+^ channel, voltage-gated Ca^2+^ channels and ERK1/2 [44–46]. Although several mechanisms have been discovered by which Gβγ activates ERK1/2, the transactivation of EGFR pathway appears to be widely explored and accepted [43, 46–48]; the same has been demonstrated for GPER in breast cancer cells [25, 41, 42]. The data presented here demonstrate that the Gβγ inhibitor, gallein, further increased GPER-mediated coronary artery relaxation, but completely blocked GPER-mediated enhancement of ET-1-induced vasoconstriction, indicating that Gβγ signaling is involved in GPER-mediated mechanisms for potentiating vasoconstriction. We speculate that activation of GPER induced both artery relaxation and constriction responses simultaneously, although the primary effect is relaxation. The direct measurement of HB-EGF release indicated that gallein blocked the HB-EGF release by G-1 treatment, further confirming the notion that Gβγ is involved in GPER-mediated transactivation of EGFR.

It is widely accepted that Src is coupled to nearly all GPCRs that lead to EGFR transactivation [49]. This notion has also been demonstrated in breast cancer cells with GPER [25]. Thus, findings in our isometric tension studies showing that the Src inhibitor, PP2, blocked the vasoconstriction effect of G-1 and strongly supporting the involvement of Src in GPER-mediated transactivation of EGFR. Consistently, G-1 stimulated HB-EGF release was blocked by Src inhibitor, PP2, as well as GM6001, an inhibitor of metalloproteinases (MMP), which cleaves HB-EGF from its membrane precursor [31, 32]. In addition, since GM6001 treatment significantly inhibited G-1-enhancement of ET-1 constriction, it seems highly likely that MMP activity is a component of the transactivation of EGFR induced by GPER activation.

**Fig 4:**
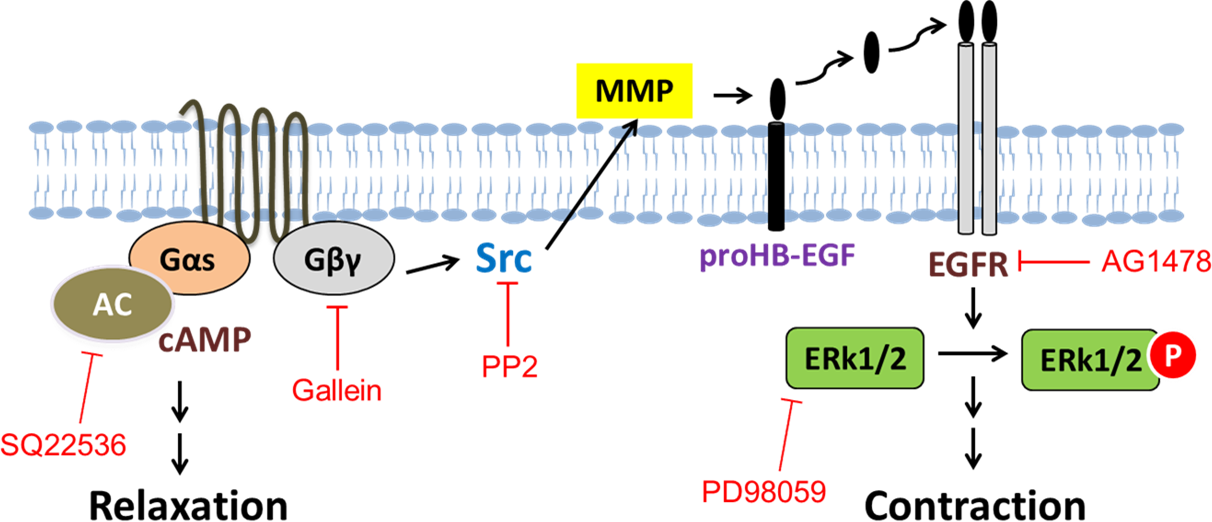
Proposed mechanism of GPER-mediated signaling in porcine coronary artery tension regulation. Activation of GPER by agonist G-1 increases cAMP production and Gβγ release, Gβγ in turn activates tyrosine kinase Src, and then metalloproteinases which cleaves and releases HB-EGF from its precursors ProHB-EGF, and HB-EGF binds and activates EGFR. Active EGFR stimulates its downstream target ERK1/2 and thus leads to contraction.

In conclusion, these studies demonstrate that the activation of GPER elicits both relaxation and contraction responses in isolated coronary arteries. Our previous work has shown that GPER also induces relaxation via cAMP [13, 14]. However, GPER-mediated potentiation of coronary artery contraction involves the transactivation of EGFR and the phosphorylation of ERK1/2 via Gβγ signaling (Fig 4). These data provide important new information that helps unravel the ongoing controversy regarding the mechanism(s) responsible for the action of estrogen to both prevent and contribute to coronary heart disease.

## References

[1] Go AS, Mozaffarian D, Roger VL, Benjamin EJ, Berry JD, Borden WB, et al Heart disease and stroke statistics—2013 update: a report from the American Heart Association. Circulation. 2013;127(1):e6 doi: 10.1161/CIR.0b013e31828124ad 23239837 10.1161/CIR.0b013e31828124ad PMC5408511

[2] Schmidt P. The 2012 hormone therapy position statement of the North American Menopause Society. Menopause (New York, NY). 2012;19(3):257. 10.1097/gme.0b013e31824b970a PMC3443956 22367731

[3] Hodis HN, Mack WJ. Hormone replacement therapy and the association with coronary heart disease and overall mortality: clinical application of the timing hypothesis. The Journal of steroid biochemistry and molecular biology. 2014;142:68–75. doi: 10.1016/j.jsbmb.2013.06.011 23851166 10.1016/j.jsbmb.2013.06.011

[4] Low AK, Russell LD, Holman HE, Shepherd JM, Hicks GS, Brown CA. Hormone Replacement Therapy and Coronary Heart Disease in Women:: A Review of the Evidence. The American journal of the medical sciences. 2002;324(4):180–4. 12385489 10.1097/00000441-200210000-00003

[5] Han G, Li F, Yu X, White RE. GPER: a novel target for non-genomic estrogen action in the cardiovascular system. Pharmacological Research. 2013;71:53–60. doi: 10.1016/j.phrs.2013.02.008 23466742 10.1016/j.phrs.2013.02.008

[6] Haas E, Bhattacharya I, Brailoiu E, Damjanovic M, Brailoiu GC, Gao X, et al Regulatory role of G protein-coupled estrogen receptor for vascular function and obesity. Circ Res. 2009;104(3):288–91. Epub 2009/01/31. doi: CIRCRESAHA.108.190892 [pii] doi: 10.1161/CIRCRESAHA.108.190892 ; PubMed Central PMCID: PMC2782532. 19179659 10.1161/CIRCRESAHA.108.190892 PMC2782532

[7] Lindsey SH, Cohen JA, Brosnihan KB, Gallagher PE, Chappell MC. Chronic treatment with the G protein-coupled receptor 30 agonist G-1 decreases blood pressure in ovariectomized mRen2. Lewis rats. Endocrinology. 2009;150(8):3753–8. doi: 10.1210/en.2008-1664 19372194 10.1210/en.2008-1664 PMC2717873

[8] Broughton BR, Miller AA, Sobey CG. Endothelium-dependent relaxation by G protein-coupled receptor 30 agonists in rat carotid arteries. American Journal of Physiology-Heart and Circulatory Physiology. 2010;298(3):H1055–H61. doi: 10.1152/ajpheart.00878.2009 20061543 10.1152/ajpheart.00878.2009

[9] Meyer MR, Baretella O, Prossnitz ER, Barton M. Dilation of epicardial coronary arteries by the G protein-coupled estrogen receptor agonists G-1 and ICI 182,780. Pharmacology. 2010;86(1):58–64. doi: 10.1159/000315497 20639684 10.1159/000315497 PMC3201835

[10] Yu X-Y, Mu C-L, Gu C, Liu C, Liu X-J. Impact of woodchip biochar amendment on the sorption and dissipation of pesticide acetamiprid in agricultural soils. Chemosphere. 2011;85(8):1284–9. doi: 10.1016/j.chemosphere.2011.07.031 21862101 10.1016/j.chemosphere.2011.07.031

[11] Yu X, Ma H, Barman SA, Liu AT, Sellers M, Stallone JN, et al Activation of G protein-coupled estrogen receptor induces endothelium-independent relaxation of coronary artery smooth muscle. Am J Physiol Endocrinol Metab. 2011;301(5):E882–8. doi: 10.1152/ajpendo.00037.2011 ; PubMed Central PMCID: PMC3213995. 21791623 10.1152/ajpendo.00037.2011 PMC3213995

[12] Kurt AH, Buyukafsar K. Vasoconstriction induced by G1, a G-protein-coupled oestrogen receptor1 (GPER-1) agonist, in the isolated perfused rat kidney. European journal of pharmacology. 2013;702(1):71–8. 23376418 10.1016/j.ejphar.2013.01.020

[13] Yu X, Li F, Klussmann E, Stallone JN, Han G. G protein-coupled estrogen receptor 1 mediates relaxation of coronary arteries via cAMP/PKA-dependent activation of MLCP. Am J Physiol Endocrinol Metab. 2014;307(4):E398–407. doi: 10.1152/ajpendo.00534.2013 . 25005496 10.1152/ajpendo.00534.2013

[14] Yu X, Zhang Q, Zhao Y, Schwarz BJ, Stallone JN, Heaps CL, et al Activation of G protein-coupled estrogen receptor 1 induces coronary artery relaxation via Epac/Rap1-mediated inhibition of RhoA/Rho kinase pathway in parallel with PKA. PLoS One. 2017;12(3):e0173085 doi: 10.1371/journal.pone.0173085 . 28278256 10.1371/journal.pone.0173085 PMC5344336

[15] Batenburg WW, Jansen PM, van den Bogaerdt AJ, Danser AH. Angiotensin II–aldosterone interaction in human coronary microarteries involves GPR30, EGFR, and endothelial NO synthase. Cardiovascular research. 2012:cvs016. 10.1093/cvr/cvs016 22260839

[16] Bhattacharya I, Damjanović M, Dominguez AP, Haas E. Inhibition of activated ERK1/2 and JNKs improves vascular function in mouse aortae in the absence of nitric oxide. European journal of pharmacology. 2011;658(1):22–7. doi: 10.1016/j.ejphar.2010.09.053 20868664 10.1016/j.ejphar.2010.09.053

[17] Filardo EJ, Quinn JA, Frackelton AR Jr, Bland KI. Estrogen action via the G protein-coupled receptor, GPR30: stimulation of adenylyl cyclase and cAMP-mediated attenuation of the epidermal growth factor receptor-to-MAPK signaling axis. Molecular endocrinology. 2002;16(1):70–84. doi: 10.1210/mend.16.1.0758 11773440 10.1210/mend.16.1.0758

[18] Feng J, Liu Y, Khabbaz KR, Hagberg R, Sodha NR, Osipov RM, et al Endothelin-1-induced contractile responses of human coronary arterioles via endothelin-A receptors and PKC-alpha signaling pathways. Surgery. 2010;147(6):798–804. doi: 10.1016/j.surg.2009.11.016 ; PubMed Central PMCID: PMCPMC2875281. 20079914 10.1016/j.surg.2009.11.016 PMC2875281

[19] Chamley-Campbell JH, Campbell GR, Ross R. Phenotype-dependent response of cultured aortic smooth muscle to serum mitogens. The Journal of Cell Biology. 1981;89(2):379–83. 7251658 10.1083/jcb.89.2.379 PMC2111686

[20] Li F, Yu X, Szynkarski CK, Meng C, Zhou B, Barhoumi R, et al Activation of GPER Induces Differentiation and Inhibition of Coronary Artery Smooth Muscle Cell Proliferation. PLoS One. 2013;8(6):e64771 doi: 10.1371/journal.pone.0064771 ; PubMed Central PMCID: PMCPMC3686788. 23840305 10.1371/journal.pone.0064771 PMC3686788

[21] Li F, Yu X, Szynkarski CK, Meng C, Zhou B, Barhoumi R, et al Activation of GPER induces differentiation and inhibition of coronary artery smooth muscle cell proliferation. 2013. 10.1371/journal.pone.0064771 PMC3686788 23840305

[22] Bologa CG, Revankar CM, Young SM, Edwards BS, Arterburn JB, Kiselyov AS, et al Virtual and biomolecular screening converge on a selective agonist for GPR30. Nat Chem Biol. 2006;2(4):207–12. doi: 10.1038/nchembio775 . 16520733 10.1038/nchembio775

[23] Dennis MK, Field AS, Burai R, Ramesh C, Petrie WK, Bologa CG, et al Identification of a GPER/GPR30 antagonist with improved estrogen receptor counterselectivity. J Steroid Biochem Mol Biol. 2011;127(3–5):358–66. doi: 10.1016/j.jsbmb.2011.07.002 ; PubMed Central PMCID: PMCPMC3220788. 21782022 10.1016/j.jsbmb.2011.07.002 PMC3220788

[24] Overland AC, Insel PA. Heterotrimeric G proteins directly regulate MMP14/membrane type-1 matrix metalloprotease: a novel mechanism for GPCR-EGFR transactivation. J Biol Chem. 2015;290(16):9941–7. doi: 10.1074/jbc.C115.647073 ; PubMed Central PMCID: PMCPMC4400366. 25759388 10.1074/jbc.C115.647073 PMC4400366

[25] Filardo EJ, Quinn JA, Bland KI, Frackelton AR Jr. Estrogen-induced activation of Erk-1 and Erk-2 requires the G protein-coupled receptor homolog, GPR30, and occurs via trans-activation of the epidermal growth factor receptor through release of HB-EGF. Molecular endocrinology. 2000;14(10):1649–60. doi: 10.1210/mend.14.10.0532 11043579 10.1210/mend.14.10.0532

[26] Nakao F, Kobayashi S, Mogami K, Mizukami Y, Shirao S, Miwa S, et al Involvement of Src family protein tyrosine kinases in Ca2+ sensitization of coronary artery contraction mediated by a sphingosylphosphorylcholine-Rho-kinase pathway. Circulation research. 2002;91(10):953–60. 12433841 10.1161/01.res.0000042702.04920.bf

[27] Lucchesi PA, Sabri A, Belmadani S, Matrougui K. Involvement of metalloproteinases 2/9 in epidermal growth factor receptor transactivation in pressure-induced myogenic tone in mouse mesenteric resistance arteries. Circulation. 2004;110(23):3587–93. doi: 10.1161/01.CIR.0000148780.36121.47 . 15557365 10.1161/01.CIR.0000148780.36121.47

[28] Lekontseva ON, Rueda-Clausen CF, Morton JS, Davidge ST. Ovariectomy in aged versus young rats augments matrix metalloproteinase-mediated vasoconstriction in mesenteric arteries. Menopause. 2010;17(3):516–23. doi: 10.1097/gme.0b013e3181c91f04 . 20142791 10.1097/gme.0b013e3181c91f04

[29] Makki N, Thiel KW, Miller FJ. The epidermal growth factor receptor and its ligands in cardiovascular disease. International journal of molecular sciences. 2013;14(10):20597–613. doi: 10.3390/ijms141020597 24132149 10.3390/ijms141020597 PMC3821633

[30] Iwasaki H, Eguchi S, Ueno H, Marumo F, Hirata Y. Endothelin-Mediated Vascular Growth Requires p42/p44 Mitogen-Activated Protein Kinase and p70 S6 Kinase Cascades via Transactivation of Epidermal Growth Factor Receptor 1. Endocrinology. 1999;140(10):4659–68. doi: 10.1210/endo.140.10.7023 10499523 10.1210/endo.140.10.7023

[31] Lucchesi PA, Sabri A, Belmadani S, Matrougui K. Involvement of metalloproteinases 2/9 in epidermal growth factor receptor transactivation in pressure-induced myogenic tone in mouse mesenteric resistance arteries. Circulation. 2004;110(23):3587–93. doi: 10.1161/01.CIR.0000148780.36121.47 15557365 10.1161/01.CIR.0000148780.36121.47

[32] Amin AH, Abd Elmageed ZY, Partyka M, Matrougui K. Mechanisms of myogenic tone of coronary arteriole: Role of down stream signaling of the EGFR tyrosine kinase. Microvasc Res. 2011;81(1):135–42. doi: 10.1016/j.mvr.2010.11.001 ; PubMed Central PMCID: PMCPMC3022328. 21067705 10.1016/j.mvr.2010.11.001 PMC3022328

[33] Griol-Charhbili V, Fassot C, Messaoudi S, Perret C, Agrapart V, Jaisser F. Epidermal growth factor receptor mediates the vascular dysfunction but not the remodeling induced by aldosterone/salt. Hypertension. 2011;57(2):238–44. doi: 10.1161/HYPERTENSIONAHA.110.153619 . 21199998 10.1161/HYPERTENSIONAHA.110.153619

[34] Hao L, Du M, Lopez-Campistrous A, Fernandez-Patron C. Agonist-induced activation of matrix metalloproteinase-7 promotes vasoconstriction through the epidermal growth factor-receptor pathway. Circ Res. 2004;94(1):68–76. doi: 10.1161/01.RES.0000109413.57726.91 . 14656925 10.1161/01.RES.0000109413.57726.91

[35] Nagareddy PR, Chow FL, Hao L, Wang X, Nishimura T, MacLeod KM, et al Maintenance of adrenergic vascular tone by MMP transactivation of the EGFR requires PI3K and mitochondrial ATP synthesis. Cardiovasc Res. 2009;84(3):368–77. doi: 10.1093/cvr/cvp230 . 19578070 10.1093/cvr/cvp230

[36] Belmadani S, Palen DI, Gonzalez-Villalobos RA, Boulares HA, Matrougui K. Elevated epidermal growth factor receptor phosphorylation induces resistance artery dysfunction in diabetic db/db mice. Diabetes. 2008;57(6):1629–37. doi: 10.2337/db07-0739 ; PubMed Central PMCID: PMCPMC2758606. 18319304 10.2337/db07-0739 PMC2758606

[37] Benter IF, Yousif MH, Griffiths SM, Benboubetra M, Akhtar S. Epidermal growth factor receptor tyrosine kinase-mediated signalling contributes to diabetes-induced vascular dysfunction in the mesenteric bed. Br J Pharmacol. 2005;145(6):829–36. doi: 10.1038/sj.bjp.0706238 ; PubMed Central PMCID: PMCPMC1576192. 15852031 10.1038/sj.bjp.0706238 PMC1576192

[38] Nagareddy PR, MacLeod KM, McNeill JH. GPCR agonist-induced transactivation of the EGFR upregulates MLC II expression and promotes hypertension in insulin-resistant rats. Cardiovasc Res. 2010;87(1):177–86. doi: 10.1093/cvr/cvq030 . 20110336 10.1093/cvr/cvq030

[39] Dennis MK, Burai R, Ramesh C, Petrie WK, Alcon SN, Nayak TK, et al In vivo effects of a GPR30 antagonist. Nat Chem Biol. 2009;5(6):421–7. Epub 2009/05/12. doi: nchembio.168 [pii] doi: 10.1038/nchembio.168 ; PubMed Central PMCID: PMC2864230. 19430488 10.1038/nchembio.168 PMC2864230

[40] Debortoli AR, Rouver WDN, Delgado NTB, Mengal V, Claudio ERG, Pernomian L, et al GPER modulates tone and coronary vascular reactivity in male and female rats. J Mol Endocrinol. 2017;59(2):171–80. doi: 10.1530/JME-16-0117 . 28733475 10.1530/JME-16-0117

[41] Filardo EJ, Quinn JA, Frackelton AR Jr., Bland KI. Estrogen action via the G protein-coupled receptor, GPR30: stimulation of adenylyl cyclase and cAMP-mediated attenuation of the epidermal growth factor receptor-to-MAPK signaling axis. Mol Endocrinol. 2002;16(1):70–84. doi: 10.1210/mend.16.1.0758 . 11773440 10.1210/mend.16.1.0758

[42] Quinn JA, Graeber CT, Frackelton AR Jr., Kim M, Schwarzbauer JE, Filardo EJ. Coordinate regulation of estrogen-mediated fibronectin matrix assembly and epidermal growth factor receptor transactivation by the G protein-coupled receptor, GPR30. Mol Endocrinol. 2009;23(7):1052–64. doi: 10.1210/me.2008-0262 ; PubMed Central PMCID: PMCPMC2703602. 19342448 10.1210/me.2008-0262 PMC2703602

[43] Luttrell LM, Daaka Y, Lefkowitz RJ. Regulation of tyrosine kinase cascades by G-protein-coupled receptors. Curr Opin Cell Biol. 1999;11(2):177–83. . 10209148 10.1016/s0955-0674(99)80023-4

[44] Syrovatkina V, Alegre KO, Dey R, Huang XY. Regulation, Signaling, and Physiological Functions of G-Proteins. J Mol Biol. 2016;428(19):3850–68. doi: 10.1016/j.jmb.2016.08.002 ; PubMed Central PMCID: PMCPMC5023507. 27515397 10.1016/j.jmb.2016.08.002 PMC5023507

[45] Khan SM, Sleno R, Gora S, Zylbergold P, Laverdure JP, Labbe JC, et al The expanding roles of Gbetagamma subunits in G protein-coupled receptor signaling and drug action. Pharmacol Rev. 2013;65(2):545–77. doi: 10.1124/pr.111.005603 . 23406670 10.1124/pr.111.005603

[46] Pierce KL, Tohgo A, Ahn S, Field ME, Luttrell LM, Lefkowitz RJ. Epidermal growth factor (EGF) receptor-dependent ERK activation by G protein-coupled receptors: a co-culture system for identifying intermediates upstream and downstream of heparin-binding EGF shedding. J Biol Chem. 2001;276(25):23155–60. doi: 10.1074/jbc.M101303200 . 11290747 10.1074/jbc.M101303200

[47] Prenzel N, Zwick E, Daub H, Leserer M, Abraham R, Wallasch C, et al EGF receptor transactivation by G-protein-coupled receptors requires metalloproteinase cleavage of proHB-EGF. Nature. 1999;402(6764):884–8. doi: 10.1038/47260 10622253 10.1038/47260

[48] Koch WJ, Hawes BE, Allen LF, Lefkowitz RJ. Direct evidence that Gi-coupled receptor stimulation of mitogen-activated protein kinase is mediated by G beta gamma activation of p21ras. Proc Natl Acad Sci U S A. 1994;91(26):12706–10. ; PubMed Central PMCID: PMCPMC45508. 7809106 10.1073/pnas.91.26.12706 PMC45508

[49] Kopetz S. Targeting SRC and epidermal growth factor receptor in colorectal cancer: rationale and progress into the clinic. Gastrointest Cancer Res. 2007;1(4 Suppl 2):S37–41. ; PubMed Central PMCID: PMCPMC2666842. 19360146 PMC2666842

